# Lipopolysaccharide-induced hemolysis: Evidence for direct membrane interactions

**DOI:** 10.1038/srep35508

**Published:** 2016-10-19

**Authors:** Stephan Brauckmann, Katharina Effenberger-Neidnicht, Herbert de Groot, Michael Nagel, Christian Mayer, Jürgen Peters, Matthias Hartmann

**Affiliations:** 1 Klinik für Anästhesiologie und Intensivmedizin, Universitätsklinikum Essen, Universität Duisburg-Essen, Germany; 2 Institut für Physiologische Chemie, Universitätsklinikum Essen, Universität Duisburg-Essen, Germany; 3 Institut für Physikalische Chemie, Universität Duisburg-Essen, Germany

## Abstract

While hemolysis in patients with sepsis is associated with increased mortality its mechanisms are unknown and Toll-like receptor (TLR)-4 mediated effects, complement-mediated hemolysis, or direct cell membrane effects are all conceivable mechanisms. In this study, we tested the hypotheses that toxic lipopolysaccharide (LPS) as well as non-toxic RS-LPS evokes hemolysis (1) by direct membrane effects, and (2) independent of the complement system and TLR-4 activation. We found, that incubation with LPS resulted in a marked time and concentration dependent increase of free hemoglobin concentration and LDH activity in whole blood and washed red cells. Red cell integrity was diminished as shown by decreased osmotic resistance, formation of schistocytes and rolls, and a decrease in red cell membrane stiffness. Non-toxic RS-LPS inhibited the LPS-evoked increase in TNF-α concentration demonstrating its TLR-4 antagonism, but augmented LPS-induced increase in supernatant hemoglobin concentration and membrane disturbances. Removal of plasma components in washed red cell assays failed to attenuate hemolysis. In summary, this study demonstrates direct physicochemical interactions of LPS with red cell membranes resulting in hemolysis under *in vitro* conditions. It might thus be hypothesized, that not all effects of LPS are mediated by TLR and may explain LPS toxicity in cells missing TLR.

During gram-negative sepsis, lipopolysaccharide (LPS), the main constituent of the bacterial cell membrane, binds to the Toll-like receptor (TLR)-4 and activates the innate immune and hemostatic systems^1^. Subsequently, generalized inflammation and activation of coagulation evoke disturbances of the microcirculation, ischemia, and multiorgan failure often resulting in the patients‘ death^1^. TLR-4 antagonists decrease serum cytokine concentrations and mortality in experimental endotoxinemia, highlightening the importance of TLR-4 in endotoxinemia^2^,^3^. However, in a recent study the TLR-4 inhibitor eritoran failed to improve survival in patients with sepsis^4^. It can be hypothesized that this finding is due to the fact that TLR-4 has both beneficial and harmful effects in sepsis: while TLR-4 is necessary for the host defence, a generalized activation might be detrimental. An inhibition of pathophysiologic events caused by lipopolysaccharides, which are unrelated to host dependence might therefore be an attractive target for sepsis therapy. Indeed, we and others recently demonstrated that hemolysis in both humans and animals, carries a poor prognosis^5^,^6^,^7^, possibly mediated by the toxicity of hemoglobin and its degradation products^8^. Along this line, greater haptoglobin concentrations were associated with improved survival^9^. Moreover, acetaminophen administration, a potent inhibitor of hemoglobin mediated lipid peroxidation, increased survival^6^,^10^. However, while the detrimental effects of free hemoglobin become increasingly obvious, the mechanisms evoking hemolysis in sepsis are unknown.

Accordingly, we tested the hypotheses that LPS-induced hemolysis (1) is complement or cytokine-dependent, and (2) can be triggered by direct membrane interactions, without co-stimulatory mediators required.

## Results

### Time- and LPS concentration-dependent changes in supernatant hemoglobin concentration and LDH activity in human whole blood

**Figure 1:**
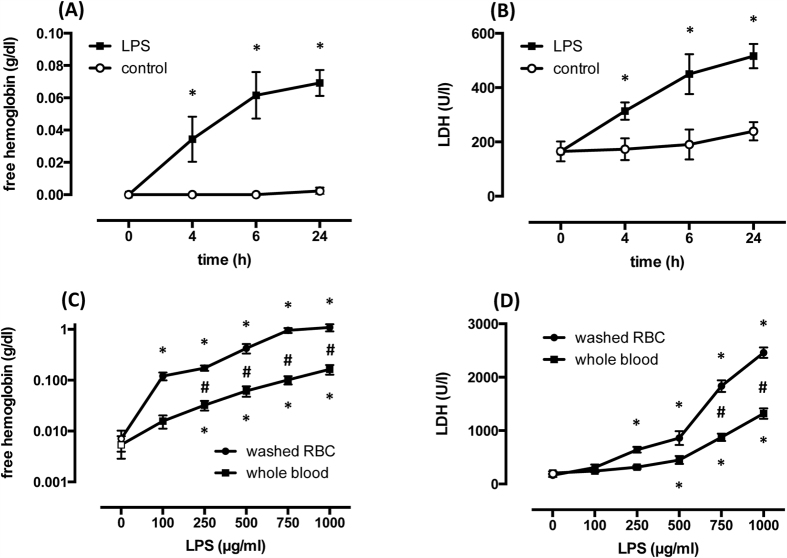
LPS (500 μg/ml) induces a time-dependent increase of free hemoglobin concentration (**A**) and LDH activity (**B**) in whole blood. Dose-dependent effect of LPS on free hemoglobin concentration (**C**) please note logarithmic scale) and LDH activity (**D**) in whole blood and washed red cells (RBC) assays during incubation for 6 h. Means ± SD; n = 12 (**C,D**) independent samples from the same donors). *p < 0.05 vs. control, ^#^p < 0.001 washed red cells vs. whole blood.

LPS (500 μg/ml) but not saline evoked a time related increase in supernatant hemoglobin concentration as well as LDH activity, as assessed by measurements at baseline and after 4, 6, and 24 h, and increased from 0.002 g/dl ± 0.001 to 0.069 ± 0.008 (n = 12, p < 0.0001; Fig. 1A) and from 239 U/l ± 34 to 516 ± 44 (n = 12, p < 0.0001; Fig. 1B), respectively, after 24 h. Since most of the increases occurred within the initial 6 hours, we chose a 6 h period in subsequent experimental series to assess the relationship between hemoglobin concentration/LDH activity and LPS concentration.

In whole blood samples exposed to increasing concentrations of LPS (100, 250, 500, 750, or 1000 μg/ml) but not in controls, free hemoglobin concentrations and LDH activity significantly increased with each LPS concentration in an apparently concentration dependent manner (Fig. 1C/D). Maximum free hemoglobin concentration in response to LPS (1000 μg/ml) averaged 0.164 g/dl ± 0.035 (n = 12, p < 0.0001; Fig. 1C). Maximum LDH activity in response to LPS (1000 μg/ml) averaged 1319 U/l ± 97 (n = 12, p < 0.0001; Fig. 1D).

### LPS-induced increase in hemoglobin concentration and LDH activity in washed red cell suspensions

To investigate LPS-induced hemolysis and LDH activity in the absence of plasmatic blood components, i.e., diminishing major contributions to hemolysis of the complement system and leukocytes, whole blood samples were removed of their buffy coat and their plasma by several centrifugation and wash steps, filtered using a leukocyte depletion filter, and red cells were subsequently resuspended in 0.9% NaCl with the hematocrit adjusted. Washed red cells were then incubated with identical LPS concentrations as used in the whole blood experiments. Notably, the increase in hemoglobin concentration was greater at each LPS concentration, and free hemoglobin concentration was about 6 times greater in washed red cells and amounted to a maximum of 1.087 g/dl ± 0.177 vs. 0.014 ± 0.003 after 6 h (n = 12; p = 0.0001; Fig. 1C). The increase in LDH activity was also greater with increasing LPS concentration, and LDH activity was about 1.8 fold higher in washed red cells and amounted to a maximum of 2460 U/l ± 98 after 6 h (n = 12; p < 0.0001; Fig. 1D).

### Effects of LPS and RS-LPS on red cell integrity and supernatant TNF-α concentration

**Figure 2:**
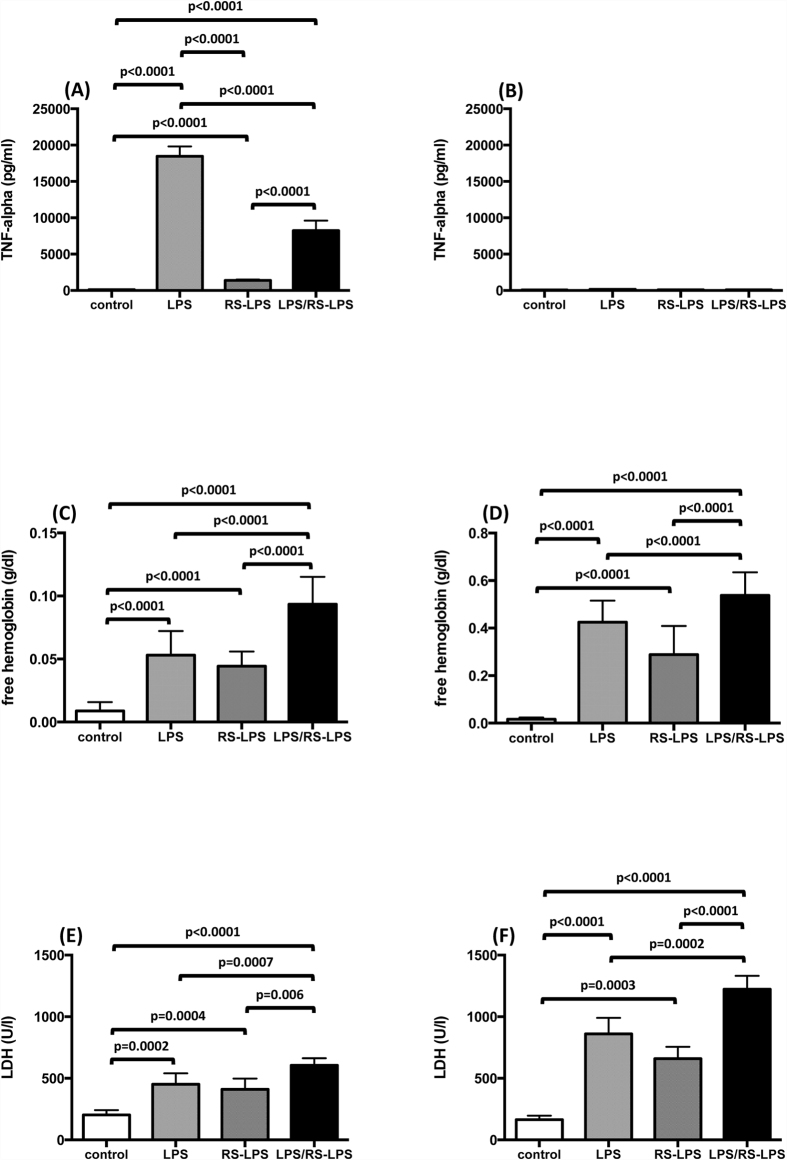
Endotoxin-induced supernatant TNF-α concentration (**A,B**) free hemoglobin concentration (**C,D**) and LDH activity (**E,F**) in whole blood (**A,C,E**) and washed red cell suspensions (**B,D,F**) during LPS (500 μg/ml), RS-LPS (500 μg/ml), and LPS + RS-LPS (500 μg/ml each) incubation for 6 h. Untreated samples served as controls. Means ± SD; n = 12.

To determine whether any LPS effect on red cell integrity is related to TLR-4 signaling, we compared the effects of LPS, the prototypic TLR-4 agonist, to those of RS-LPS, a non-toxic LPS and TLR-4 receptor antagonist, both on red cell integrity and TNF-α supernatant concentrations. LPS induced a marked increase in TNF-α concentrations in whole blood samples (121 pg/ml ± 5 to 18474 ± 1303; n = 12; p < 0.0001). TNF-α concentration in response to RS-LPS averaged 1384 pg/ml ± 114 (n = 12; p < 0.0001) (Fig. 2A). During co-incubation of 500 μg/ml LPS and 500 μg/ml RS-LPS, RS-LPS markedly diminished the LPS-induced increase in TNF-α concentration (8244 pg/ml ± 1310; n = 12; p < 0.0001), demonstrating its TLR-4 antagonistic action.

In contrast to the attenuation by RS-LPS of the LPS evoked increase of TNF-α supernatant concentration, the effect of RS-LPS on red cell integrity closely resembled that of LPS. Identical concentrations of LPS and RS-LPS evoked comparable and significant increases of free supernatant hemoglobin concentration (0.053 g/dl ± 0.019 and 0.044 ± 0.011; n = 12, p < 0.0001 and p < 0.0001) and LDH activity (452 U/l ± 82 and 411 ± 81; n = 12; p = 0.0002 and p = 0.0004) (Fig. 2C,E). Moreover, co-incubation of whole blood with both lipopolysaccharides resulted in an even greater increase in free hemoglobin concentration (0.093 g/dl ± 0.022; n = 12; p < 0.0001) and LDH activity (606 U/l ± 58; n = 12; p = 0.0007) than either LPS alone.

To confirm our findings in whole blood and to exclude plasma components inducing hemolysis, we also measured supernatant TNF-α concentration and hemolysis in washed and filtered red cells. As expected following depletion of white blood cells, no LPS-induced TNF-α response was detected (Fig. 2B).

Supernatant free hemoglobin concentrations as well as LDH activity were significantly increased after endotoxin stimulation compared to controls (Fig. 2D/F). Free hemoglobin concentration in response to LPS averaged 0.425 g/dl ± 0.091 (n = 12; p < 0.0001 vs. control; Fig. 2D) and free hemoglobin concentration in response to RS-LPS was 0.228 g/dl ± 0.121 (n = 12; p < 0.0001 vs. control; Fig. 2D). The combination of both endotoxins yielded a significant increase in free hemoglobin concentration compared to negative controls as well as to either RS-LPS or LPS alone, and the free hemoglobin concentration in response to LPS + RS-LPS averaged 0.538 pg/ml ± 0.097 (n = 12; p < 0.0001 vs. control; Fig. 2D). Likewise, LDH activity in response to LPS averaged 861 U/l ± 129 (n = 12; p < 0.0001 vs. control; Fig. 2F) and in response to RS-LPS was 659 U/l ± 97 (n = 12; p = 0.0003 vs. control; Fig. 2F). The combination of both endotoxins yielded a significant increase in LDH activity (1223 U/l ± 109, n = 12; p < 0.0001 vs. control; Fig. 2F) when compared to controls as well as to incubation with either LPS or RS-LPS alone.

### LPS and RS-LPS-induced effects on red cell osmotic resistance

**Figure 3:**
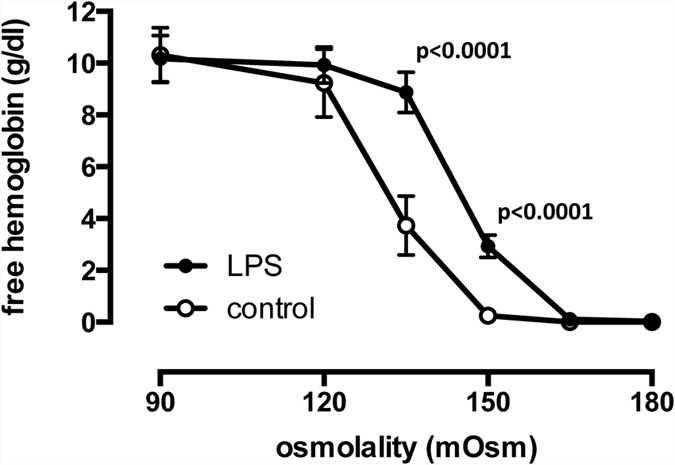
Osmotic resistance of red cells following incubation with LPS (500 μg/ml) for 6 hours in whole blood samples and vehicle controls. LPS induced a significant shift in EC_50_ from 132.3 mOsm ± 1.0 to 143.4 mOsm ± 0.9, indicating less osmotic stress tolerance. Means ± SD; n = 12.

To determine the effects of LPS and RS-LPS on red cell membrane integrity, red cell osmotic resistance was determined when incubated with either LPS, RS-LPS, or vehicle (Fig. 3). The EC_50_, i.e., the osmolality eliciting half maximum hemolysis, of LPS-treated red cell assays (143.4 mOsm ± 0.9; n = 12; p < 0.0001) was significantly increased compared to controls (132.3 mOsm ± 1.0; n = 12). Similarly, the EC_50_ of RS-LPS-treated red cells (144.3 mOsm ± 0.3; n = 12; p < 0.0001) was significantly increased compared to controls but did not differ from the EC_50_ of the LPS-treated red cells.

### LPS and RS-LPS-induced alterations in red cell morphology and volume

**Figure 4:**
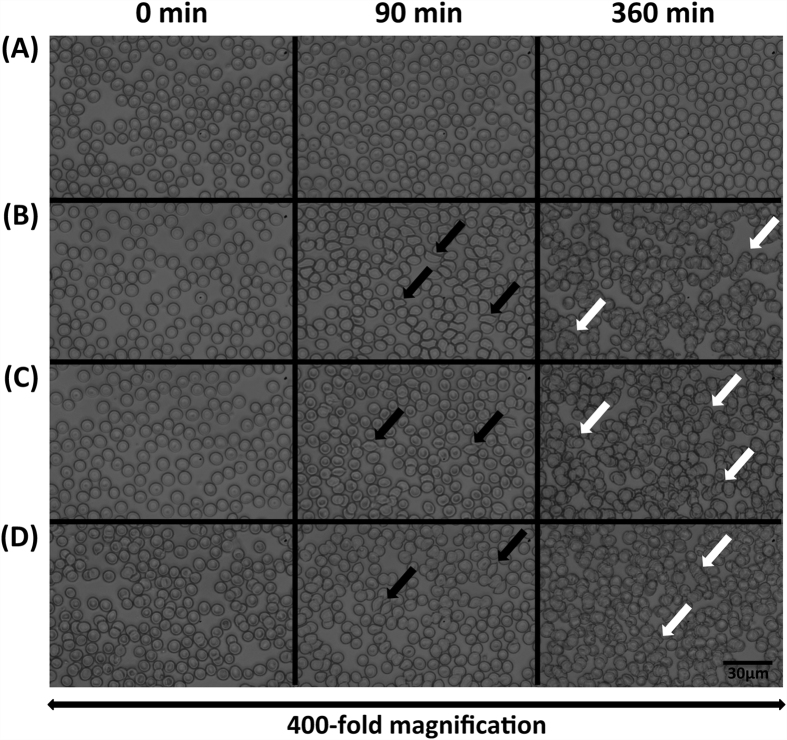
Time-dependent alterations of red cell morphology and shape after incubation with vehicle (**A**) LPS (500 μg/ml) (**B**) RS-LPS (500 μg/ml) (**C**) and combined LPS + RS-LPS (500 μg/ml each) (**D**) as shown in blood smears assessed by light microscopy. Data from representative experiments. Black arrows: schistocytes, white arrows: pseudo-agglutination (rouleaux formation). No morphological changes were observed in controls (**A**).

Whole blood was incubated with LPS (500 μg/ml), RS-LPS (500 μg/ml), LPS and RS-LPS combined (500 μg/ml each), or vehicle, and red cell morphology was investigated by light microscopy. After 90 min of incubation, schistocyte formation (up to 20% of red cells) was detected after incubation with LPS, RS-LPS, or their combination (Fig. 4B–D) but not in controls (Fig. 4A). After 360 min of incubation, red cell shape appeared irregular and rouleaux formation was observed (Fig. 4B–D).

Mean red cell volume following incubation with LPS (97.0 fl ± 1, n = 6) and RS-LPS (97.1fl ± 0.7, n = 6) did not significantly change compared to controls (96.3 fl ± 0.81, n = 6).

### LPS and RS-LPS-induced alterations in red cell membrane stiffness

**Figure 5:**
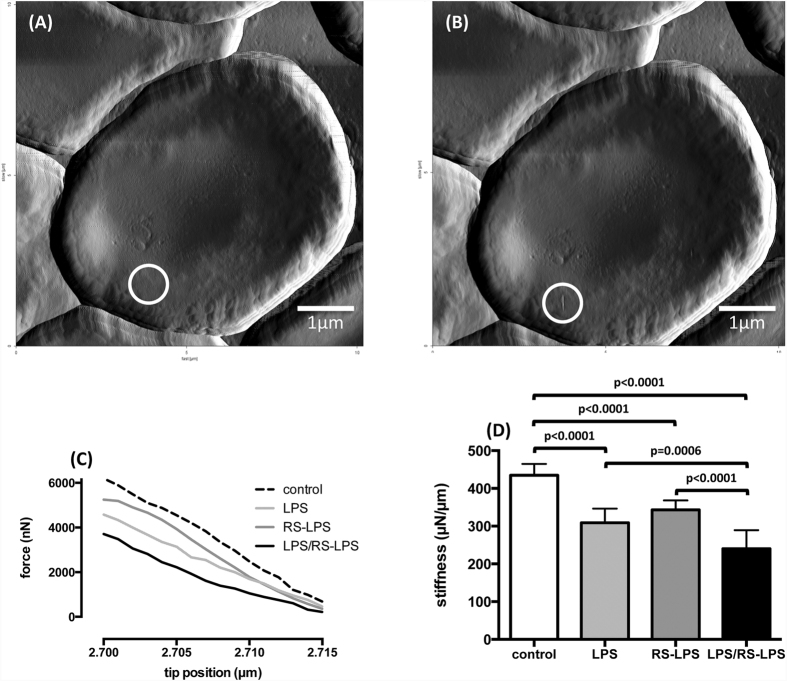
Representative images of an individual human red cells before (**A**) and after (**B**) determination of membrane stiffness by atomic force microscopy. White circle in the images indicate the region selected for the measurement. A persistent membrane indentation is seen after the measurement. (**C**) Data from a representative experiment with the vertical force plotted vs. the actual tip position. (**D**) Data from all experiments (means ± SD; n = 12).

Red cell membrane stiffness was assessed by the AFM nanoindentation technique following incubation for 6 h with LPS (500 μg/ml), RS-LPS (500 μg/ml), LPS and RS-LPS combined (500 μg/ml each), and vehicle, respectively, as exemplified and shown in Fig. 5A–D. Membrane stiffness decreased in LPS-treated red cells compared to controls (308 μN/μm ± 35 vs. 434 ± 30, n = 12, p < 0.0001). Membrane stiffness following RS-LPS decreased to 342 μN/μm ± 24 (n = 12, p < 0.0001 vs. control). Co-incubation with both endotoxins decreased membrane stiffness to 240 μN/μm ± 47 (n = 12, p < 0.0001 vs. control), and this decrease in stiffness was greater than with LPS or RS-LPS alone.

## Discussion

Our data demonstrate for the first time, that LPS induces both hemolysis in isolated washed red cells and direct effects on their cell membrane, as shown by decreased osmotic resistance, diminished membrane stiffness, and schistocyte formation. Notably, RS-LPS, a non-toxic LPS from *R. sphaeroides*, induced hemolysis to a degree comparable to LPS, both in the presence and absence of plasma components. Moreover, hemolytic action of LPS and RS-LPS combined was greater than the action of either LPS alone. Since TLR-4 antagonistic action of RS-LPS was confirmed in our system by diminution of supernatant TNF-α concentration and hemolysis also occurred in washed red cells, i.e., with an absent complement system, TLR-4 signaling as well as involvement of the complement system can be excluded as primary mechanisms of LPS-induced hemolysis. These results strongly suggest that, at least *in vitro*, hemolysis in whole blood as well as in washed red cells is evoked by direct, receptor-independent interaction of endotoxins with the red cell membrane itself, and without the requirement of additional proteins or co-stimulatory mediators. Direct membrane effects of LPS were further supported by demonstration of abnormalities in membrane characteristics under a variety of experimental settings.

The intercalation of LPS with lipid membranes is well known^11^,^12^,^13^. We recently showed, that LPS molecules quickly insert into phospholipid bilayers, increase membrane fluctuation amplitudes, and significantly weaken their mechanical stiffness^14^. In experiments with LPS from *K. pneumonia*, *S. enterica*, and *E. coli*, Ciesielski *et al*. reported direct interactions of LPS with artificial lipid bilayers, by high LPS binding capacities in raft-containing cholesterol-rich membranes^12^. Spontaneous incorporation of LPS into giant unilamellar vesicles generating shape changes and vesicle fission were also detected^11^. The authors pointed out that insertion of LPS into leukocyte membranes‘ external monolayer triggered the formation of lipid microdomains and that, therefore, endotoxin molecules can vertically diffuse to their receptors leading so as to activate TLR-4 signal cascade. Thus, direct interaction of LPS with the red cell membrane and consecutive impairment of cell integrity, as shown by our data, is supported by other evidence.

No information is available on mechanisms evoking hemolysis in endotoxinemia. In our study, we used supernatant free hemoglobin concentration as well as LDH activity as convenient markers for hemolysis in whole blood and washed red cell preparations^15^. After LPS exposure, both free hemoglobin concentration and LDH activity comparably increased. It is obvious, therefore, that cell damage and hemolysis occurred concomitantly. Accordingly, LPS, in a time and concentration-dependent manner, induces membrane disturbances and hemolysis in both whole blood and washed red cell suspensions. Such a direct membrane effect of LPS in mediating hemolysis is supported by several lines of experimental evidence:

First, LPS-induced hemolysis increased in washed red cell suspensions in comparison to whole blood, despite the removal of plasma components. This may be explained by increased binding of LPS to red cell membranes when plasma proteins are absent. Complement-induced hemolysis triggered by LPS can be excluded as a mechanism of hemolysis in our *in vitro* system since depletion of plasma by washing of the red cells prior to incubation with LPS did not mitigate hemolysis.

Furthermore, contribution to hemolysis of TLR-4 and innate immune system activation was excluded by the ability of RS-LPS to increase supernatant free hemoglobin concentration and LDH activity, but to suppress the LPS-induced increase in supernatant TNF-α concentration. Since the magnitude of hemolysis with LPS and RS-LPS was comparable at identical concentrations this is further evidence for the similar physicochemical characteristics of both molecules to evoke hemolysis via membrane action. Moreover, increased RS-LPS-induced hemolysis was also observed in washed red cells, showing striking similarities in the membrane effects of LPS and RS-LPS.

Finally, we demonstrated a direct membrane effect of LPS since red cell membrane stiffness decreased; a decrease in red cell volume as the cause was excluded. This is in-line with an increased viscosity of the lipid part of red cell membranes from both clinical and experimental septic models without alterations in mean corpuscular volume or mean corpuscular hemoglobin concentration^16^. Furthermore, we show that both LPS and RS-LPS decrease stress tolerance of the red cell membrane.

Potential limitations of our study might include the use of *in vitro* systems. Indeed, some mechanisms potentially evoking hemolysis like disseminated intravascular coagulation were not investigated, so as not to allow interference of direct membrane effects addressed in this study with other mechanisms so as not to blur direct LPS effects on red cells. Conversely, however, due to its physicochemical nature any direct membrane effect of LPS is likely to prevail also *in vivo*. The use of large LPS concentrations in our assays might be considered a limitation since Mitaka *et al*. recently showed an average LPS plasma concentration of 16 pg/ml in septic patients^17^. This plasma concentration, however, is unlikely to prevail in cell membranes. According to measurements of Poeschl *et al*.^18^ in red cells incubated with LPS (concentration 1 mg/ml), only 25.5 μg LPS/ml was contained in the red cell membrane but three times more (77 μg LPS/ml) in red cell membranes obtained from patients with gram-negative sepsis. Therefore, supernatant LPS concentrations used in our study are suitable to mimic clinical concentrations at the site of action and serum LPS concentrations in septic patients do not correlate with total endotoxin concentrations^18^. Rather, its chemical structure likely allows LPS to penetrate lipid membranes of various cell types.

LPS evoked hemolysis in sepsis is associated with increased mortality^5^,^6^,^10^. Potential mechanisms include activation of the innate immune system by extracellular hemoglobin synergistic to LPS^19^,^20^, nitric oxide scavenging by free hemoglobin inhibiting NO-induced vasodilation evoking microvascular perfusion disturbances^21^, radical formation with modifications of lipids, proteins, and DNA, inflammation by iron release^22^, as well as other mechanisms^23^,^24^,^25^. In turn, free hemoglobin toxicity is counteracted by evolutionary conserved detoxification pathways including hemoglobin binding by haptoglobin, CD163 mediated uptake of the complex, binding of heme to hemopexine with complex degradation in the liver, and iron binding by the ferritin/transferrin system^26^.

It is important to state that endotoxinemia is not restricted to infection. For example, release of LPS from the gut has been described in cardiac surgery as well as various other settings with malperfusion and might contribute to hemolysis^27^.

Our data demonstrate important physicochemical interactions of LPS with the red cell membrane, irrespective of plasma constituents and TLR-4 signaling, evoking red cell membrane impairment and hemolysis. These findings are important for several reasons and lead to important questions. 1. The inhibition of free hemoglobin effects as a TLR-4 independent noxious mechanism of LPS might be a good candidate for sepsis therapy as innate immune system function important for host defense is likely not altered. 2. Most probably, incorporation of LPS is not limited to red cell membranes but will occur in every cell type: incorporation apart from red cells thus could activate signal transduction with important consequences for the organism. 3. LPS incorporated in cell membranes might serve as a depot for LPS; the fate of LPS in this compartment is unknown and warrants further attention. 4. The diagnostic value of free hemoglobin and red cell membrane characteristics, respectively, in patients with sepsis should be compared in further studies.

## Materials and Methods

LPS- (*Escherichia coli* serotype 0111:B4; Sigma-Aldrich, Saint Louis, USA) induced lysis of red cells was investigated by measuring free hemoglobin concentration in whole blood preparations. To assess any contribution of the complement system experiments were repeated in washed red cell assays. The impact of TLR-4 signaling was investigated by incubation of red cells with the non-toxic RS-(*Rhodobacter sphaeroides*) LPS (InvivoGen, San Diego, USA), a TLR-4 receptor antagonist, both alone and in combination with *E. coli* LPS. The action of LPS on red cell membrane properties was determined by measurements of osmotic resistance, light microscopy, and atomic force microscopy (AFM).

### Preparation of human whole blood, washed red cells, and endotoxin exposures

The study was reviewed and approved by the Ethics Committee at the Medical Faculty of the University of Duisburg-Essen. All methods were performed in accordance with the ethical guidelines and regulations, and informed consent was obtained from all participating individuals. Citrated whole blood of healthy donors was withdrawn via puncture of a medial cubital vein. Mean hematocrit was 39–45%. To prepare washed red cell samples, cells were isolated by centrifugation (3000 *g*, 15 min), washed twice in 0.9% NaCl with buffy coat removal after each centrifugation step, and leukocyte depleted filter (BioR, Fresenius Kabi, Bad Homburg, Germany). Subsequent, cells were resuspended in 0.9% NaCl and hematocrit was adjusted to comparable values (mean 39–45%).

Both whole blood and washed red cell samples were immediately stimulated with endotoxins, i.e., LPS, RS-LPS, or their combination (LPS + RS-LPS), in various concentrations (0, 100, 250, 500, 750, or 1000 μg/ml), and incubated at 37 °C for up to 24 h. After incubation, supernatants were collected and stored at −80 °C until measurements of free hemoglobin concentration, plasma activity of lactate dehydrogenase (LDH), and tumor necrosis factor (TNF)-α concentration. Red cells were collected for immediate light as well as atomic force microscopy, respectively, and measurements of cell volume and osmotic resistance.

### Red cell morphology

Peripheral blood smears were prepared by the wedge slide technique. To assess red cell morphology light microscopy was performed on an inverted microscope (Zeiss IM35, Carl Zeiss AG, Oberkochen, Germany) using a magnification of 400×. Images were taken using a Canon 1000D single lense reflex camera (Canon, Krefeld, Germany). Changes in red cell morphology were compared with time-related controls without LPS exposition. Definition of schistocytes and rolls was according to the International Council for Standardization in Hematology (ICSH) and quantified as the percentage of undamaged red cells with 1,000 counted cells^28^,^29^.

### Assessment of red cell membrane stiffness

Red cell membrane stiffness was measured using the atomic force microscopy nanoindentation technique (“Nano Wizard”, JPK Instruments, Berlin, Germany) and non-contact/tapping mode high resonance frequency (NCH) cantilevers (NanoWorld, Neuchatel, Switzerland). An intermittent contact mode at a frequency of 240–270 kHz was used for the imaging. The scan rate was adjusted to 0.75 Hz. The tip was horizontally positioned over the red cell’s convex peripheral border and brought in contact with the cell membrane. During the force-tapping mode, the cantilever holder was moved vertically over a distance of 200 nm towards the carrier surface within 5 s. Force mapping was conducted with cantilevers and spring constants of approx. 42 N/m. The cantilever deformation was used to calculate the actual tip position subject to the individual force applied to the red cell membrane.

### Red cell volume and leukocyte count

To determine mean red cell volume in endotoxin-stimulated and unstimulated control assays and exclude relevant volume changes potentially altering their membrane stiffness, and to validate sufficient removal of leukocytes from washed red cells, a Coulter Counter (Sysmex CA 500, Sysmex GmbH, Norderstedt, Germany) was used.

### Assessment of red cell osmotic resistance

To assess red cell osmotic resistance, whole blood samples preincubated with LPS (500 μg/ml) or vehicle for 6 h were incubated for 10 minutes in solutions of different ionic strength, as obtained by serial dilution of NaCl 0.9% with endotoxin-free bidestilled water. Thereafter, supernatant free hemoglobin concentration was determined and the EC_50_ of the relationship between free hemoglobin concentration and sodium concentration was derived by curve fitting (GraphPad Prism Software Inc., San Diego, USA).

### Cell free hemoglobin and TNF-α concentrations, and LDH activity

Free supernatant hemoglobin concentration was measured spectrophotometrically from the absorption of the hemoglobin *Soret* band (μQuant, Bio-Tek Instruments GmbH, Friedrichshall, Germany)^30^. Supernatant LDH activity was determined with a clinical chemistry analyzer (Vitalab Selectra E, VWR International, Darmstadt, Germany). Supernatant TNF-α concentration was measured using a sandwich ELISA kit (R&D Systems GmbH, Wiesbaden-Nordenstadt, Germany) and specific monoclonal antibodies according to the manufacturer’s protocol.

### Materials

All chemical agents were of analytical grade and endotoxin-free bidestilled water and NaCl 0.9% were used (B. Braun Melsungen AG, Melsungen, Germany).

### Data analysis and statistics

Data are presented as mean and standard deviation (SD) unless stated otherwise. Analyses were performed using SPSS 18.0 (SPSS Inc., Chicago, IL) and GraphPad Prism 6 (GraphPad Prism Software Inc., San Diego, CA). Since the D’Agostino-Pearson normality test revealed a normal distribution of values of variables statistical analysis was performed using one-way or two-way analysis of variance, as appropriate, with post-hoc Bonferroni correction. An a priori alpha error p of less than 0.05/n was considered statistically significant.

## Additional Information

**How to cite this article**: Brauckmann, S. *et al*. Lipopolysaccharide-induced hemolysis: Evidence for direct membrane interactions. *Sci. Rep.* **6**, 35508; doi: 10.1038/srep35508 (2016).
